# Low-fat, lactose-free and leucine-enriched chocolate cow milk prototype: A preliminary study on sensorial acceptability and gastrointestinal complaints following exhaustive exercise

**DOI:** 10.1186/s12970-020-00406-0

**Published:** 2021-02-10

**Authors:** Cristiano D. da Silva, Dirce R. de Oliveira, Ítalo T. Perrone, Carlos H. Fonseca, Emerson S. Garcia

**Affiliations:** 1 Department of Physical Education, Institute of Life Sciences, Federal University of Juiz de Fora, Campus: Governador Valadares, Rua Manoel Byrro, 241 - Vila Bretas, Governador Valadares, MG 35010-260 Brazil; 2 School of Physical Education, Physiotherapy and Occupational Therapy, Federal University of Minas Gerais, Belo Horizonte, MG Brazil; 3 Department of Basic Life Sciences Institute of Life Sciences , Federal University of Juiz de Fora , Campus Governador Valadares, MG Governador Valadares, Brazil; 4 Department of Food Engineering, Center of Exact Sciences, Federal University of Viçosa, Viçosa, MG Brazil; 5 Pharmaceutical Department, Faculty of Pharmacy, Federal University of Juiz de Fora, Campus Juiz de Fora, MG Brazil; 6 Pharmaceutical Department, Institute of Life Sciences, Federal University of Juiz de Fora, Campus Governador Valadares, MG Brazil; 7 Department of Physical Education, Federal University of Maranhão, São Luís, Maranhão Brazil

**Keywords:** Soccer player, Athlete, Discomfort, Sports nutrition, Product acceptability, Workout recovery, Milk-based beverage

## Abstract

**Background:**

Chocolate milk has gained recent scientific support as a recovery drink. However, it is known that high exercise-demand triggers gastrointestinal discomfort which continues post-exercise, thereby hindering this nutritional strategy. In addition, those who are lactose intolerant cannot benefit from a milk-based beverage. Thus, the aim of this preliminary study was to develop a low-fat, lactose-free, and leucine-enriched chocolate cow milk prototype (CML) representing nutrition-related recommendations for football players, as well as assess athletes’ individual subjective outcomes for gastrointestinal complaints and sensorial acceptability in a field-based setting following strenuous team-sport physical demands.

**Methods:**

This study followed a single group and repeated-measured design with 10 football players (23 ± 2 yrs., 74 ± 14 kg, 174 ± 5 cm) who consumed CML following a 90-min football match simulation protocol (FMP). The total CML intake to achieve 0.150 g leucine·kg [BW]·h^− 1^ occurred in aliquots of 50, 30 and 20% at 0-, 45- and 75-min post-FMP, respectively. Athletes were evaluated by the prevalence, the type and severity (bloating, nausea, flatulence, and gastric reflux) of gastrointestinal complaints and sensorial acceptability (overall perception, appearance, consistency, and flavour) after drinking each aliquot in a 4-h recovery period.

**Results:**

The CML showed higher scores for “Product Acceptability Index” (88%) and sensorial acceptability (~ 8 in 9-point hedonic scale). Kendall’s W with bootstrapped resample (95%CI) revealed agreement among respondents as “moderate” (overall perception, flavour) to “strong” (appearance, consistency) and with no significant agreement differences between rater response in the timeline analysis (0.57 up to 0.87; *p* > 0.05). Agresti-Caffo *add-4* analysis (95% confidence interval, [95%CI]) revealed no differences in each time-point analysis versus baseline for athletes classified as having severe gastrointestinal symptoms, but confirmed concern with bloating (three athletes showed a transient response at 2-h and only one continued until 3-h; *p* = 0.051).

**Conclusions:**

These preliminary findings suggest that CML presents good taste and high acceptability by the sampled athletes. Thus, CML may be an alternative sport drink for immediate post-workout supplementation to overcome the energy deficit, offer co-ingested leucine, maintain palatability and adherence including lactose intolerance following a team sport-specific fatigue.

**Trial registration:**

RBR-2vmpz9, 10/12/2019, retrospectively registered.

## Introduction

Nutrition is considered one of sports performance pillars, and fulfilling the post-exercise nutrition-related recommendations is fundamental for the recuperative and adaptive process effectiveness. Hence, an effective recovery strategy between workouts or inside competitive events may maximize an adaptive response to a variety of fatigue-mechanisms improving muscle structure and functionality and thereby increasing exercise tolerance. In addition to enabling better adaptability conditions, an effective recovery intervention can also gradually make an athlete’s body become better for particular features of sport demands which are important to performance and injury prevention. Thus, an effective nutritional recovery intervention taking into account the food supply timing and quality is considered fundamental [1, 2].

Current post-workout nutrition-related recommendations for multiple sprint sports athletes point to the offer of a carbohydrate-protein mixture (CHO:PRO) [2–4]. The need for this CHO:PRO combined intervention is justified by previous evidence regarding an increased muscle glycogen storage, muscle damage amelioration, and greater acute and chronic training adaptations in the above mentioned sport-workload context [2]. By addressing such mechanisms in detail, it is evident that the CHO:PRO mixture improves the acute recovery process via stimulation of muscle protein synthesis (MPS), as well as the activation of the target mechanism of rapamycin (*mTOR*), signalling a pathway [5, 6] and a more effective glycogen storage via insulinotropic response [7, 8].

Considering CHO:PRO natural mixtures and other potential nutrients, milk and chocolate milk have recently gained scientific support as recovery drinks in adults [9–11] and adolescent athletes [12]. Despite the promising aspect of chocolate milk as a recovery drink, those lactose intolerants will not be able to benefit from this strategy. In addition, athletes may experience gastrointestinal discomfort and reduced appetite after high-demand sports activities [13, 14]. In this sense, there is still a lack of evidence regarding the use of chocolate milk beverages and prevalence of gastrointestinal discomfort, observing conflicting results [10, 15]. Furthermore, chocolate milk may be a high palatability supplement, but few studies have tested its sensorial acceptability [16].

Exercise scientists, support professionals, and athletes may have a strong interest in special formulation alternatives to conventional sports drinks, such as chocolate milk, which are designed to meet a high metabolic cost in exhaustive team sports. Another reason for the interest is to find an alternative beverage proposal which does not compromise adherence to the continuity recovery diet plan due to gastrointestinal distress as demonstrated in commercial recovery aid [16] or because high rate of diuresis impairing rehydration as occurred in post-workout plans using conventional sports drinks [11, 13]. In this context, we developed a low-fat, lactose-free, and leucine-enriched chocolate cow milk prototype (CML). With regard to this unprecedented experimental sport-specific beverage, the aim of this preliminary study was to propose an empirical clarification producing the CML, testing its supplementation use and analysing athletes’ individual subjective outcomes on gastrointestinal complaints and sensorial acceptability. In the trial, we proposed a practical (“ready-to-drink”) and functional intake protocol (lactose-free and representing nutrition-related recommendations for football players), and maintained similar conditions to real-life use following a football-specific acute fatigue.

## Methods

### Experimental design

This study was preliminary and focused on individual subjective outcomes, and therefore it provides guidelines to support a large-scale randomized controlled trial in the future. Moreover, a real consumer’s emotional state (i.e. post-exhaustive exercises) was considered appropriate to simulate a real beverage consumption, constituting a context which could compromise the acceptance and sensory rejection of the product [17]. Therefore, a repeated measures independent group was adopted, and a randomized or placebo-controlled trial was not considered adequate in this preliminary study. As an attempt to make the study more controllable, athletes did not concomitantly intake water or other foods with CML consumption, avoiding apparent bias in subjective outcomes, which are the focus of this study. Researchers were not involved in participant recruitment or any assessments.

In the experimental phase, the study began with a particular CML proposition and process (see the beverage composition and processing proposal below). Athletes had to report to the laboratory on two different occasions. The first visit preceded the main trial to sample descriptive characterization, individual habitual food intake data for prescribing a pre-trial meal and beverage intake (e.g., leucine intake and ingestion volume), and conduct trial familiarization with a football-match simulation protocol. The second visit involved a pre-trial meal and standardized hydration; baseline data; fatiguing exercise via a standardized football match-related protocol; CML ingestion; and post-intake data in the recovery period timeline. The outcome measures of interest were gastrointestinal complaints (7 within-effect measurement times) and taste (3 within-effect measurement times) within a 4 h follow-up period.

### Participants

Ten male (23 ± 2 yrs., 74 ± 14 kg, 174 ± 5 cm) university-standard football outfield players (defenders [*N* = 3]; midfielders [*N* = 5]; attackers [*N* = 2]) were recruited from a randomization procedure in the team squad to participate in this study. The subjects were informed of the purpose, procedures, and associated risks prior to signing an informed written consent obtained according to a protocol approved by the Research Ethics Committee of Federal University of Minas Gerais, Brazil (CAAE: 32153514.5.0000.5149). Subjects finished their competitive season (of the University Athletic League) approximately 2 weeks before the study and were engaged in unstructured training to keep fit. Players trained 4–5 days per week on the field (technical-tactical) during the season, including 2 conditioning-based sessions (i.e., high-intensity training, sprinting, strength/plyometric work) and one match. Health and well-being were prioritized, thus the sample size and all subjects in this preliminary study were not lactose intolerant, nor allergic to dairy products, with these same subjects being interviewed in the sample selection stage.

### Beverage composition and processing

The CML prototype was prepared approximately 1 week before consumption using a cow milk and dairy product platform for mixing the ingredients until homogeneous and then pasteurization. Once ready, the CML was kept at refrigerator temperature and tested according to Brazilian food regulations and microbiological conditions for milk and dairy products - IN 16/2005 [18]. Food microbiology analyses were performed using Petrifilm 3 M™ methodologies (3 M Company, St. Paul/MN, USA), in agreement with standards of Brazilian specific legislation - RDC 12/2001 [19].

In the sport-specific beverage formulation, the first order was to consider the ~ 3:1 CHO:PRO ratio (nutritional content per 200 mL = 204 kcal; 35 g [CHO]; 12 g [PRO] and 3.3 g [FAT]) to simulate chocolate milks presented in previous studies [9–11] and nutrition-related recommendations [2, 20] to formulate a more effective recovery drink to be used immediately after game or trainings in football players. In addition, functional enrichment in the beverage was thought to improve its amino acid profile. Thus, the CML would enable synergistic effects in the recovery process or physical stress adaptation, evidenced by the use of protein mixture, leucine hydrolysates, and CHO in insulin response amplification [21–24]. Although naturally present in cow’s milk, essential amino acids (EAAs) are critically needed for achieving maximal rates of MPS, requiring high quality protein sources (which contain EAAs, including leucine) [20]. Therefore, the CML and its use were designed to offer a continuous supply of amino acids in circulation, suggested at the design conception time to be at a leucine intake of ~ 0.150 g.kg.[BW]^− 1^ or ~ 10 g.h [BW]^− 1^ [22, 23, 25] and 6–20 g of EAA [24, 26]. Leucine intake of CML was approximately 70% below the tolerable upper limit [27, 28].

### Procedures

In the first visit, the athletes were characterized (weight and height) and familiarized using a standardized 90-min football-match simulation protocol (FMP) [29, 30]. The energetic value of the athletes’ habitual intake was determined by registering their usual food diet during three consecutive days, and also including their average weekend consumption [31]. We calculated their habitual macronutrient consumption using a nutritional software analysis (Avanutri®, Rio de Janeiro, Brazil). At the end of the first visit procedures, they were asked to abstain from alcohol consumption and to moderate strenuous activity 24 h in advance, as well as caffeine-containing products for 12 h prior to the trial. The athletes were required to keep a 3-day written record of their habitual food intake.

**Fig. 1:**
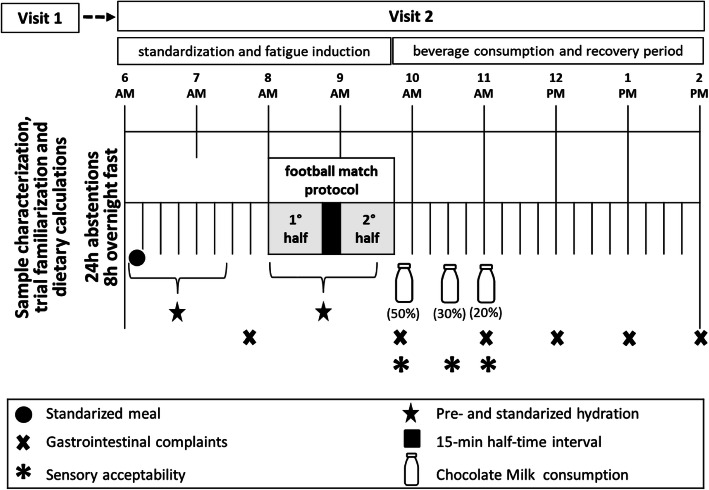
Schematic diagram for the study protocol timeline including visit requirements, fatigue induction via a standardized 90-min football match simulation protocol (FMP), measurements flow, treatment with experimental chocolate cow milk beverages and recovery period

On the second visit, the athletes arrived at the laboratory in the morning following an overnight fast (8 h). A urine sample was collected from each athlete to measure and compare their isovolumetric water quantities before and during FMP. Then, all athletes received a pre-trial standardized meal (~ 380 kcal, 68 g of CHO, 11 g of PRO, 7 g of FAT) and 5 mL.kg [BW]^− 1^ of water [32]. Athletes classified into hypohydrate state (Urine Specific Gravity; USG ≥ 1.020 a.u.) [33] received an additional 2 mL.kg [BW]^− 1^ of water [34]. After the meal, the athletes rested for 75 min and were then invited to wear the appropriate clothing and footwear to perform 15 min of standard football match-day warm-up and stretching. The hydration in FMP occurred with water (2 mL.kg [BW]^− 1^) every 15 min up to 75 min to ensure adequate hydration [32]. FMP was a 90-min fatiguing exercise protocol (T-SAFT^90^) corresponding to an official football match played from audio commands [29, 30]. Each player used a strap equipment component of Polar Team System® (Polar Electro Oy, Kempele, Finland). FMP was conducted on a natural grass football pitch at 25 ± 1 °C and 63 ± 1% RH. The athletes were referred for CML supplementation and passive recovery at the end of the FMP. The athletes were placed in thermoneutral room (23 ± 1 °C, 60 ± 1 RH) during and after the pre-FMP meal, as well during the treatment and recovery period. A scheme of the study protocol timeline and collection plan of each variable is shown in Fig. 1.

### CML treatment and recovery period

The athletes remained in the laboratory for 4 h of relaxed recovery following the FMP, where they received the total volume of CML for treatment (~ 630 mL) in aliquots at three separate moments: 50% at time 0, 30% at 45 min and 20% at 75 min. This hypothetical-deductive CML intake plan aimed to increase adherence to treatment (appetite appeal and less satiety, and so, ingest the final treatment volume), and especially to allow a continuous supply of circulating amino acids and insulinemic response. Athletes were seated and instructed to drink the entire offered volume quota in 5 min. A lab technician was responsible for the timing, scheduling, and delivery of beverage. The beverage was served at 4 °C.

### Subjective measures

A questionnaire was conducted in pre-trial (baseline), immediately post-FMP and every hour up to 4-h post-intake for evaluating the type and severity of gastrointestinal symptoms (bloating, nausea, flatulence, and gastric reflux) on a scale of “1” to “10” arbitrary unit (a.u.), with “1” referring to “no symptoms” and “10” to “severe symptoms” [35].

Sensory acceptability was evaluated in the following attributes: overall perception, appearance, consistency, and flavour. Athletes were requested to rate the drink according to a 9-point hedonic scale from “1” (“I did not like it very much”) to “9” (“I really liked it”) [36]. The equation [PAI% = average grade obtained for the product * 100/maximum grade given to the product] was adopted to calculate the “Product Acceptability Index” (PAI) [37].

### Data analysis

Sample description and intake volume were presented with means ± sd. Gastrointestinal complaint severity were divided into two categories: severe symptoms and less severe symptoms [35]. Prevalence of severe symptoms were only registered when a score of “5” or higher out of “10” a.u. was given. Less severe symptoms were registered when a score below “5” was given. Taste and overall perception were presented as frequency of each sensory attribute. Friedman rank sum test was used to verify the sensorial acceptability time effect. Comparisons of proportions of athletes with severe gastrointestinal symptoms in each time point versus baseline was calculated using the multivariate normal distribution (Dunnett-adjusted). Standard normal quantiles and estimates with 95% confidence interval (95% CI) were calculated by the Agresti-Caffo *add-4* [38]. The degree of sensorial acceptability agreement between responders from each aliquot intake was analysed using Kendall’s W coefficients agreement degree (0 ≤ W ≤ 1, 1 representing perfect concordance) with bootstrapped reanalysis (95% CI; creating 1000 bootstrap from the original data). All statistical analyses were performed using R software environment for statistical computing (R Core Team, version 3.6.0). The statistical significance was set at *p* < 0.05 for all analyses.

## Results

The metabolic load impact variables of the FMP were: exercise intensity (85 ± 7%HR_peak_), body mass change (− 2.3 ± 0.9%) and USG (1018 ± 6 a.u.). According to the subjects’ characteristics (23 ± 2 yrs., 74 ± 14 kg, 174 ± 5 cm) and individual desired dose of leucine ingestion, the CML average intake was 625 ± 61 mL (312 ± 31 mL at time 0; 187 ± 18 mL at 45 min and 125 ± 12 mL at 75 min post-FMP, respectively). The total caloric intake average was 637 ± 62 kcal.

Most athletes (70%) did not report any severe gastrointestinal symptoms in 4-h of treatment and recovery period (Table 1). However, a transient effect was observed 2-h post-intake on three athletes (30%) who reported severe scores for bloating. Two of these three previously mentioned athletes presented concomitant nausea and gastric reflux, and the one other volunteer presented flatulence. Comparisons of proportions of athletes with severe gastrointestinal symptoms revealed no differences in each time-point analysis versus baseline, but confirmed concern with bloating symptom (Agresti-Coull *add-4* analysis [− 0.26, 0.43, 95%CI]; *p* = 0.051; Table 1). Only one volunteer who presented “bloating” had a follow-up of this symptom 3-h post-intake. No participant abandoned this study due to gastrointestinal distress.

**Table 1:** Prevalence of severe gastrointestinal complaints during experimental chocolate cow’s milk supplementation

Complaint	post-FMP	1-h post-intake	2-h post-intake	3-h post-intake	4-h post-intake	*p-value*
bloating	0 [0.0; − 0.29, 0.29]	0 [0.0; − 0.29, 0.29]	3(30%) [0.3; − 0.16, 0.66]	1(10%) ^b^ [0.1; − 0.26, 0.43]	0 [0.0; − 0.29, 0.29]	0.051
nausea	0 [0.0; − 0.29, 0.29]	0 [0.0; − 0.29, 0.29]	2(20%) ^a^ [0.2; − 0.21, 0.55]	0 [0.0; − 0.29, 0.29]	0 [0.0; − 0.29, 0.29]	0.127
flatulence	0 [0.0; − 0.29, 0.29]	0 [0.0; − 0.29, 0.29]	1(10%) ^a^ [0.1; − 0.26, 0.43]	0 [0.0; − 0.29, 0.29]	0 [0.0; − 0.29, 0.29]	0.833
gastric reflux	0 [0.0; −0.29, 0.29]	0 [0.0; − 0.29, 0.29]	2(20%) ^a^ [0.2; − 0.21, 0.55]	0 [0.0; − 0.29, 0.29]	0 [0.0; − 0.29, 0.29]	0.127

Results are given as absolute and relative prevalence values, n (%) [estimates; lower and upper confidence limits with 95% confidence interval] calculated by the Agresti-Caffo *add-4* CI (Agresti & Caffo, 2000) [38] considering the difference of proportions in each time point versus baseline condition using the multivariate normal distribution (Dunnett) standard normal quantiles. Prevalence was registered when a score of “5” or higher arbitrary unit (out of “10”) was informed. *P-value* calculated from the maximum of test statistics with unpooled variance estimators. Prevalence criterion used was supported by Jeukendrup et al. (2000) [35]. FMP = football match simulation protocol. ^a^concomitance with bloating. ^b^follow-up symptom

Quantitative analyses via the Friedman test showed that there was no significant difference for the aliquot time effect for all of the attributes’ acceptance (*p* > 0.05). The qualitative results of the sensory acceptability for CML revealed that most athletes (60–80%) answered as “I really liked it” and “really enjoyed it” (Table 2), with the degree of agreement among responders observed as “moderate” (overall perception, flavour) to “strong” (appearance, consistency), and with no significant agreement differences between the raters’ responses in the different evaluation times (Kendall’s W agreement degree: 0.57 up to 0.87; *p* > 0.05).

**Table 2:** Sensorial acceptability of experimental chocolate cow’s milk

Attribute acceptance	Observed answers	1st aliquot	2nd aliquot	3rd aliquot	Aliquot effect and agreement degree	
					*x*^2^_2_ (*p-value*)	Kendall’s W (95% CI)
overall perception	“I really liked it”	1(10%)	1(10%)		0.800 (0.670)	0.57 (0.53, 1)
	“I really enjoyed it”	7(70%)	7(70%)	8(80%)		
	“I moderately liked it”	2(20%)	2(20%)	1(10%)		
	“I slightly liked it”			1(10%)		
appearance	“I really liked it”	2(20%)	3(30%)	2(50%)	0.667 (0.716)	0.87 (0.85, 1)
	“I really enjoyed it”	6(60%)	4(40%)	5(50%)		
	“I moderately liked it”					
	“I slightly liked it”	2(20%)	3(30%)	3(30%)		
consistency	“I really liked it”	2(20%)	3(30%)	3(30%)	0.000 (1.000)	0.81 (0.79, 1)
	“I really enjoyed it”	6(60%)	4(40%)	4(40%)		
	“I moderately liked it”	2(20%)	2(20%)	2(20%)		
	“I slightly liked it”		1(10%)	1(10%)		
flavor	“I really liked it”	2(50%)	1(10%)	2(20%)	0.737 (0.692)	0.63 (0.59, 1)
	“I really enjoyed it”	5(50%)	5(50%)	4(40%)		
	“I moderately liked it”	3(30%)	3(30%)	3(30%)		
	“I slightly liked it”		1(10%)	1(10%)		

Results are given as absolute and relative value, n(%). Total beverage intake = 625 ± 61 mL. Aliquot intake = 50, 30 and 20% at first (0 min), second (45 min) and third (75 min) post-FMP, respectively. The sensory acceptability criterion was the 9-point hedonic scale proposed by Meilgaard et al. (2006) [36]. *x*^2^_2_, chi squared with df = 2 and *p-value* were obtained by Friedman rank sum test (used for to verify the time effect). Kendall’s W with bootstrapped 95% CI (confidence interval with bootstrap [1000 replicates recalculated from original data]) was used to describe agreement among respondents

The overall sensorial evaluation showed higher scores (~ 8 out of 9-point) for all attributes’ acceptance criterion (e.g., perception, appearance, consistency, and flavour; Table 3). The CML had a high classification score regarding the PAI (88%).

**Table 3:** Sensory attribute acceptability of experimental chocolate cow milk anchored on a 9-point hedonic scale

Sensory attributes	mean ± sd (95% CI)
Overall perception	7.8 ± 0.4 (7.5, 8.1)
Appearance	7.7 ± 0.9 (7.1, 8.3)
Consistency	7.9 ± 0.6 (7.5, 8.4)
Flavour	7.7 ± 0.7 (7.3, 8.1)

Results are given as mean ± sd and 95% CI (confidence interval with bootstrap [1000 replicates recalculated from original data]). Total beverage intake = 625 ± 61 mL. The sensory acceptability criterion was the 9-point hedonic scale proposed by Meilgaard et al. (2006) [36]

## Discussion

Post-workout supplementation strategies are an interesting topic for athletes and well-trained people. Therefore, it is necessary to formulate and test new sports drinks with compositions for particular workload recovery demand, as well as to create viable plans for use in daily sport contexts. Thus, the present study formulated a CML prototype in aiming to meet this applicability and technological gap. Furthermore, this ready-to-drink sport-specific drink was evaluated on sensorial acceptability and gastrointestinal complaint scores using football players as potential consumers in an ecological team sport workload and recovery in a real sporting setting. The FMP (adapted SAFT^90^) had similar physiological [39] or mechanical load [29] to football games, reinforcing the validity of the protocol as an instrument for fatigue induction corresponding to an official football match as demonstrated in the external validation study of this protocol [30].

In agreement with our hypotheses, the results demonstrate that the plausible short-term CML benefits do not compromise adherence to a supplementation plan in an immediate post-workout recovery timeline via severe gastrointestinal complaints or for not liking the beverage. Therefore, we believe that the results of the CML are encouraging because it indicates that athletes will not lose their adherence to the proposed supplementation plan. For example, an expressive result for CML was that the majority of athletes answered “I really liked it” or “really enjoyed it”, pointing out a higher and consistent timeline response for the product acceptability index (~ 88%) [37]. In addition, this intervention approach with multi-ingredient supply was proposed to match the physical demands which referenced-team sports could create, providing more valuable evidence for athletes to adhere to strategic recovery plan, and how it affects post-match nutrition intervention.

Although preference and gastrointestinal complaints about CML use were not tested against conventional sports drinks or commercial chocolate milk (in order to control confounding variables), and the fact that all athletes had dairy tolerance (motivated to safety), the relatively low prevalence (30%) of severe bloating symptoms observed 2-h post-intake did not extrapolate the cut-off point of five points from any gastrointestinal discomfort criteria. Thus, the observed scores did not compromise the gastrointestinal sensation, being classified as “non-severe” [35], thus making the CML results encouraging for new and expanded studies.

Leucine-enriched chocolate milk, as present in the CML prototype, is interesting for athletes because they may help preserve their skeletal muscle during various catabolic states, as well as stimulate MPS via insulin-dependent anabolic stimuli or via *mTOR* during early exercise recovery [23, 24]. Unfortunately, isolated leucine supplementation could trigger symptoms such as bloating, flatulence, and abdominal pain. Moreover, there is evidence that leucine may have some effects on specific hypothalamic-brainstem circuits which connect amino acid availability and nutrient detection to food intake control [40]. As a result, dietary leucine largely escapes first-pass metabolism, plasma leucine levels are rapidly and markedly increased, and it is likely to represent a physiological signal of hypothalamic amino acid availability [40]. Thus, the combination of chocolate cow milk with one of the most important EAAs may be a valid supplementation strategy to consider. Furthermore, the intake protocol through fractions (aliquots) made the CML intake safe, tolerable, and physiologically interesting for immediate recovery nutritional intervention.

The issues of sensory acceptability and gastrointestinal complaints are important in practice, as athletes cannot stop eating for non-compliance and for reasons due to gastrointestinal discomfort in later stages of recovery. Thus, the bloating gastrointestinal complaint impact of CML was small, considering that other conditions and workload characteristics in team players could aggravate the sensation of gastrointestinal discomfort. In this scope, the present study used a trial which simulates a football match-related fatigue via standardized protocol [29, 30]. Thus, gastrointestinal or appetite problems could be expected following this exhaustive exercise, as already presented in literature. For example, previous studies have demonstrated slow gastric emptying in football match-play settings [41] or during a football sport–specific running patterns protocol (Loughborough Intermittent Shuttle Test [LIST]) [42]. Reduced mesenteric blood flow has also previously been shown during intense exercise, especially in hypohydration condition, contributing to the development of gastrointestinal symptoms [43].

The gastrointestinal discomfort was perhaps minimized by the ingestion protocol, although it would be an expected compromise after using milk-based drinks. Cow milk proteins are known for having a low absorption rate due to gastric acid-induced coagulation. Moreover, it can also cause a transient fullness sensation because milk is rich in calcium [44]. Chocolate milk studies in literature have shown controversial results. For example, with a decreased and transiently hunger sensation compared with commercial sport drink (CHO-Electrolytes) [10, 15] or in milk-based beverage use [44]; or with no gastrointestinal complaints for appetite using commercial chocolate milk versus a commercial meal replacement beverage [16].

Due to the new product type in our study, as well as the different dose-response approach, comparisons between previous literature is very difficult, which represents a drawback in the methodology. Studies by Karp et al. [10] and Thomas et al. [15] conducted experiments with athletes being allowed to drink water ad libitum*,* which may have contributed to triggering the sensation of stomach distension. Thus, the likely transient satiation sensation may be dependent on energy density, composition, and especially intake protocol. Therefore, the gastrointestinal complaint phenomenon could transiently compromise an athlete’s gastrointestinal sensation and hunger in post-exercise phase, and CML and its intake protocol seemed to prevent these problems.

No studies were found which used sensory analysis to evaluate any similarity between leucine-supplemented milk and commercial chocolate beverages, for example. Despite this, it is possible to note that the present experimental product acceptance results and its attributes are reasonable and indicative that additional development and studies of CML could be continued. For example, CML showed higher flavour values in comparison with one study evaluating three chocolate drinks which are currently marketed in Brazil, including the market leader, using the same attributes of “overall perception” and “flavour”, presenting values at about 6 a.u [45].. However, this study evaluated sensory acceptability at post-exercise moments as done in the present study when factors such as physiological and psychological factors may cause different sensorial evaluations [37]. In addition, our study method used habitual or potential consumers in a real-world context (e.g., football players), dismissing trained tasters. Further studies and evaluations in the scope of food science and technology with physicochemical parameters are also recommended to gain a holistic understanding of drivers for liking the CML product and to continue its development to a larger scale of consumers.

In addition to the promising results of the researched CML intervention, several limitations need to be considered when interpreting its results. First, the sample was limited in size, and the one-group study design lacks a practical or ecological validity control condition. Secondly, discrepancies in composition or energy density, different solubilization rates, and the glycemic index would make the inference of results obscure (i.e., not ideal taste-matched placebo or control) if conventional sports drinks/commercial chocolate milk were used in the study protocol. In addition, the sampled athletes are users of the market leader sports drink in their usual routine in training, games, and post-workout. Thus, the authors in this study tried to avoid any previous involvement with brands and commercial products so as to avoid bias, because when consumers know certain product ingredients and expectations or when a pre-existing perception of the ingredient can influence the liking drivers [17]. We also consider that the experimental product in this study is unprecedented, and that comparisons with local chocolate drinks would not provide relevant information. After all, chocolate drinks in local legislation have distinct characteristics of product identity and would be distant from the supplementation recommendations for athletes, because they have a lot of sugar, no enrichment and loose most of the natural benefits of milk through the thermal process (UHT) and sterilization for shelf life.

Regardless of our sample characteristic, or limited hydration protocol conducted with only water before and during exhaustive exercise, it could be plausible that CML prototype and its intake protocol proved to be an attractive post-match nutrition strategy for football players. CML energy intake (~ 700 kcal) enabled meeting approximately 50% of energy requirements in an official football match in elite athletes [46], and may help to increase adherence to athletes’ nutritional plans according to the practicality for logistics in a competitive scenario. Future studies can investigate the effectiveness of milk-based beverage intake, such as CML, on metabolism, immune state and redox, postprandial fullness sensation, rehydration, performance, and appetite effect on young athletes to professional-standard players.

## Conclusion

These preliminary findings suggest that CML may be an important nutritional intervention for use in post-high-demand exercise while maintaining palatability and with no severe gastrointestinal complaints. The lactose-free CML and leucine-enriched formulation, as well as its intake protocol, could be strategies which could expand the user public as it is a safer and team-sports demands-driven approach of chocolate milk. Moreover, it can help in balancing the nutritional composition and post-workout drink effectiveness. Furthermore, some multi-ingredient characteristics, such as CML along with the presence of other nutrients such as antioxidants, amino acids, vitamins, and minerals, may be advantageous over conventional sports drinks or isolated micronutrient supplements. Additional studies are recommended to examine the proof-of-concept solution and the potential advantages of this new sport-specific drink and its mechanisms involved in recovery from fatigue from team-sports demands in elite players, including for long-term use.

## Data Availability

Please contact author for data requests.

## Abbreviations

%HR_peak_: Percentage of heart rate peak
a.u.: arbitrary unit
BW: Body weight
CHO: Carbohydrate
CI: Confidence intervals
CML: Chocolate cow milk enriched with L-leucine
FMP: Football match protocol (adapted version of SAFT^90^: soccer-specific aerobic field test)
MPS: Muscle protein synthesis
METs: Metabolic equivalents
mTOR: mammalian target of rapamycin
PRO: Protein
RH: Relative humidity
SD: Standard deviation
UHT: Ultra-high temperature
USG: Urine specific gravity
